# Articulation recovery in ALS patients after lineage-negative adjuvant cell therapy - preliminary report

**DOI:** 10.7150/ijms.47002

**Published:** 2020-07-19

**Authors:** Wioletta Pawlukowska, Bartłomiej Baumert, Monika Gołąb-Janowska, Ewa Pius-Sadowska, Zofia Litwińska, Maciej Kotowski, Agnieszka Meller, Iwona Rotter, Jarosław Peregud-Pogorzelski, Przemysław Nowacki

**Affiliations:** 1 Department of Medical Rehabilitation and Clinical Physiotherapy, Pomeranian Medical University, Szczecin, Poland.; 2 Department of General Pathology, Pomeranian Medical University, Szczecin, Poland.; 3 Department of Neurology, Pomeranian Medical University, Szczecin, Poland.; 4 Department of General Pathology, Pomeranian Medical University, Szczecin, Poland.; 5 Department of General Pathology, Pomeranian Medical University, Szczecin, Poland.; 6 Department of General Pathology, Pomeranian Medical University, Szczecin, Poland.; 7 Department of Neurology, Pomeranian Medical University, Szczecin, Poland.; 8 Department of Medical Rehabilitation and Clinical Physiotherapy, Pomeranian Medical University, Szczecin, Poland.; 9 Department of Paediatric Oncology, Pomeranian Medical University, Szczecin, Poland.; 10 Department of Neurology, Pomeranian Medical University, Szczecin, Poland.

**Keywords:** dysarthria, amyotrophic lateral sclerosis, speech disorders, LIN^-^ stem/progenitor cells, neurotrophins

## Abstract

**Background:** Amyotrophic lateral sclerosis (ALS) is one of the most frequently occurring neurodegenerative diseases affecting speech and swallowing. This preliminary study aimed to investigate whether an autologous lineage-negative stem/progenitor cell therapy applied to ALS patients affects the level of selected trophic and proinflammatory factors, and subsequently improves the articulation.

**Methods:** We enrolled 12 patients with sporadic ALS, who underwent autologous bone marrow-derived lineage negative (LIN^-^) cells administration into cerebrospinal fluid (CSF). We evaluated patients' articulation using the Frenchay Dysarthria Assessment on days 0 and 28 following the LIN^-^ cells administration. Concentrations of various factors (BDNF, NGF, ANGP-2, VEGF, PDGF-AA, PEDF, COMP-FH, CRP, C3, C4) in CSF were quantified by multiplex fluorescent bead-based immunoassays in the samples collected on the day of LIN^-^ cells administration and 28 days later. On top of this, we assessed levels of BDNF and NGF in the patients' plasma on the day of the injection, three, seven days and three months after the treatment.

**Results:** Of the 12 patients who received the LIN^-^ cell therapy 8 showed short-termed improvement in articulatory functions (group I), which was particularly noticeable in better phonation time, lips and soft palate performance, swallowing reflex and voice loudness. Four patients (group II) did not show substantial improvement. CSF concentrations of BDNF, ANGP-2 and PDGF-AA in group I decreased significantly 28 days after LIN^-^ cells administration. The highest concentration levels of BDNF in group II and NGF in both groups in blood plasma were observed on day 3 following the injection.

**Conclusions:** The outcomes of the LIN^-^ cell application in ALS treatment of articulatory organs are promising. The procedure proved to be safe and feasible. A short-lasting trophic effect of autologous LIN^-^ administration could encourage repeated cell's application in order to sustain their beneficial effects, however this approach needs further investigation.

## Introduction

Amyotrophic lateral sclerosis (ALS) is one of the most common neurodegenerative diseases leading to communication disorders, predominantly dysarthria (80-93% of ALS patients), and impaired swallowing. Disturbed speech accounts for 25-30% of ALS prodromal symptoms 1-2. Dysarthria in ALS may take 2 forms - bulbar and pseudobulbar. Speech disorders in ALS include impairments of respiration, phonation, articulation and prosody 3.

Current treatment options for speech disorders in ALS patients are limited to pharmacological intervention and clinical speech therapist support. Pyridostigmine is used to reduce muscle weakness, carbamazepine, phenytoin and lamotrigine to reduce tongue fasciculation, while atropine and amitriptyline prevent excessive salivation 4-6. Other approaches available for ALS patients with palatal flaccidity include palatal prostheses and surgical soft palate stiffening procedure, both of which aim to improve velopharyngeal competence and speech clarity 7. ALS patients often undergo clinical speech therapy. A widespread alternative utilized for speech and communication enhancement in ALS patients is Augmentative and Alternative Communication (AAC). This form of aided communication involves using an external item (picture, object, drawing) selectively used to facilitate communication between the patients and those around them 8. An important role in this area is played by a system of brain-computer interfaces (BCI), which, using sophisticated software, processes and reproduces patient's messages 9.

Despite open access to and widespread use of pharmacological means as well as dedicated speech therapy, clinical outcomes of treatments aimed at improving articulation in ALS are still far from satisfying. Due to a complex pathogenesis of the disease, the portfolio of existing medications has virtually not been changed for years. There is an urgent need for novel treatment strategies. Approaches using various subpopulations of stem cells have already shown some promise. Cell therapy is assumed to trigger a broad range of reactions within the microenvironment of the nervous tissue and promote cytoprotection of neurons 10. The administration of autologous stem/progenitor cells (SPCs) to patients with neurodegenerative disorders is expected to result in trophic support for the host's neurons, slowdown in the progression of the disease, stimulation of deficient neurotransmitters secretion, differentiation of applied cells into oligodendrocyte progenitor cells or neurons 10.

Since the 1950's, when neurotrophic factors were discovered and defined, there has been a steady increase in the research dedicated to neurotrophic protective factors, with a particular focus on: nerve growth factor (NGF), brain-derived neurotrophic factor (BDNF), neurotrophin 3 (NT-3) or neurotrophin 4 (NT-4) 11. Apart from having a crucial neuroprotective effect, BDNF also serves as a neurotransmitter modulator, plays an important role in neuronal survival and growth 12-13. There have been attempts to use its pleiotropic properties to maintain balance between the neuroregenerative and neurodegenerative processes occurring in ALS 10-12.

However, neurotrophins generally do not cross the blood-brain barrier to any substantial degree, and direct injection into neural tissue to target the effects of NTs is difficult 14. First reports regarding the effects of such a pathophysiological compilation following the delivery of brain-derived neurotrophic factor from genetically modified mesenchymal stem cells with overexpressed neurotrophins are promising 15. Other cytokines like hepatocyte growth factor (HGF), vascular endothelial growth factor (VEGF) or insulin-like growth factor 1 (IGF-1), although do not belong to the family of neurotrophic factors, exert various influence on neuronal survival, brain maturation and general functioning of the nervous system 10-11. On top of that, they regulate neuroregenerative/neurodegenerative processes and have a biological effect on stem and progenitor cells 14-16. It is also worth noting the fact that VEGF does cross the brain-blood barrier 17.

Recent studies have shown that neuroinflammation mediated by glial cells and systemic immune system activation can also contribute to the progression of ALS through both neuroprotective and neurodegenerative mechanisms 17-18. The involvement of systemic inflammatory response has also been suggested in this disease, as ALS patients presented with elevated levels of C-reactive protein and C3 and C4 complement components 17-19.

A thorough analysis of lineage negative (LIN^-^) cells in our earlier studies revealed its composition as a heterogenic population of cells consisting of precursor cells, progenitor cells and stem cells 20. The fraction did not contain any morphotic elements exhibiting mature phenotype 20. The influence of LIN^-^ cells administration on trophic factors, pro-inflammatory factors and miRNA expression in ALS patients were the subject of intensive research by our team 11. Our current study aims to analyze the articulatory functions in ALS patients and evaluate the trophic effects of autologous LIN^-^ stem/progenitor cells administration on those functions.

## Material and Methods

### Subjects

The study was designed as a prospective, open-label, nonrandomized clinical trial in a single center for patients with ALS. The trial (NCT02193893, retrospectively registered in July 18, 2014; study start date: January 2010) was approved by the Ethics Committee of the Pomeranian Medical University in Szczecin, and performed in accordance with the Declaration of Helsinki 11. Prior written informed consent was obtained from all of the subjects.

Twelve patients took part in the study - four women and eight men - aged 21 to 65 years (mean 50.33 ± 13.34) fulfilling the criteria of sporadic ALS 11 according to El Escorial Revised Criteria 21. The study included patients with a prognosis of survival over 12 months determined on the basis of general and neurological condition as below:

- mild to moderate disability documented by satisfactory bulbar and spinal motor functions (minimum score 3 on the ALS Functional Rating Scale Revised for swallowing and 2 points for food preparation and walking);
- forced vital capacity (FVC) result greater than or equal to 50%;
- without cancer, signs of inflammation, diabetes, cardiovascular disease, chronic kidney and liver disease, in euthyreosis.

LIN^-^ stem/progenitor cells were administered during the hospitalization at the Department of Neurology of the Pomeranian Medical University in Szczecin. Each time, from the moment of recruitment, a 3-month observation period was allowed for the observation of the natural course of the disease, with continuous administration of riluzole. Patients older than 65 years were obligatorily excluded from the study due to the fact that cell growth of expanded *in vitro* stem cells decreases with age 22. In addition, patients with familial form of ALS, or receiving any medication that could affect the bone marrow, were also excluded.

### Speech evaluation

#### Speech test

One of the most important tests for objective evaluation of articulation is the Frenchay Dysarthria Assessment (FDA). The FDA is a standardized test which relies on a 9-point rating scale applied to a patient. It provides information based on observations of oral structures, functions and speech. The second edition of FDA utilizes the latest findings concerning motor speech disorders and their contribution to neurological diagnosis. It has good feasibility (missing data <5 %), a high reliability of the total score (0.94), an excellent inter-rater agreement for the total score (0.96) and moderate to large construct validity for 81% of its items 23. The subjects underwent the assessment on days 0 and 28 following the LIN^-^ cells administration. Based on a detailed analysis of the FDA scores the patients were divided into 2 groups: group I, comprising patients who demonstrated improvement of at least five functions affecting the efficacy of the articulation organs and group II - patients who did not show such improvement. The test was conducted by a clinical speech therapist with 12 years' experience of treating neurological conditions, predominantly Parkinson's Disease (PD).

### Cells

Bone marrow (BM) was collected from ALS patients after obtaining their informed consent. Each time, 40-50 mL of BM samples were harvested in local anesthesia from the posterior iliac crest and subsequently resuspended in collecting medium (PBS, pH 7.2) and heparin (20 U/mL; Gibco, USA). The collected BM was lysed in BD PharmLyse Lysing Solution (BD Biosciences, San Jose, USA) for 15 min at room temperature in the dark and washed twice in phosphate-buffered saline (PBS). The procedure was performed in compliance with the manufacturer's protocol, as previously described 11. The obtained suspension of BM nucleated cells (NCs) was subjected to immunomagnetic separation (MiniMACS, Miltenyi Biotec, Auburn, USA). LIN^-^ stem/progenitor cells (mean 8.98 ± 5.77×10^6^) were isolated from non-separated NCs using immunomagnetic isolation and a Lineage Cell Depletion Kit (Miltenyi Biotec, Auburn, USA), as previously described 20. Before administration, the isolated cells were kept in 2 mL of sterile PBS.

### Administration procedure

The suspension of LIN^-^ SPCs in PBS (2 mL) was administered into the subarachnoid space by lumbar puncture between the lumbar vertebrae L3/L4 or L4/L5. LIN^-^ cells were administered to patients in both groups in similar quantities (group I: 8.99 ± 6.65×10^6^; group II: 8.98 ± 4.37×10^6^; *p=*1.00). Cell suspension was slowly injected into the subarachnoid space. Afterwards, the patients remained in a supine position for 24 to 48 hours.

### Molecular analysis - multiplex assay

Concentrations of various factors: BDNF, NGF, angiopoietin 2 (ANGP-2), VEGF, platelet-derived growth factor-AA (PDGF-AA), pigment epithelium-derived factor (PEDF), complement factor H (COMP-FH), CRP, C3, C4 in CSF were quantified by multiplex fluorescent bead-based immunoassays (Luminex Corporation, Austin, USA) in the samples collected on the day of the cell administration and 28 days later. On top of this, we assessed levels of BDNF and NGF in the patients' plasma on the day of the injection, three, seven days and three months after the treatment. The procedure was performed according to the manufacturer's protocol, as previously described 24.

### Statistical analysis

The primary objective of this investigation was to analyze the alternative hypothesis that autologous lineage-negative SPCs administered intrathecally in ALS patients do not influence the concentration of growth factors assessed in plasma and cerebrospinal fluid. To assess the equality of variances for variables the Levene's test was used before the comparison of means. The test has shown significance (*p<*0.05). For this reason and because of the non-normality of the distributions between variables (Shapiro-Wilk test), the numerical data were compared between the groups using the nonparametric Mann-Whitney U-test for variables included to the two groups. Wilcoxon matched-pairs test for two groups was also used. Friedman ANOVA and Kendall concordance were used to assess within-subjects (repeated measures) analysis of variance. *p<*0.05 was considered to indicate statistical significance. All statistical analyses were performed with STATISTICA 12.5 PL.

## Results

Comparative analysis of the average number of transplanted LIN^-^ cells in the two groups did not demonstrate statistically significant differences (*p=*1.00). Patients from group I were substantially older (*p=*0.07), the disease occurred at a later age (*p=*0.07) and had a shorter duration of the disease (*p=*0.11). **Table 1** presents characteristics of both groups.

The post-treatment examination of the articulatory functions revealed markedly improved phonation time (7 out of 8 patients), lips performance (6 out of 8 patients), swallowing reflex, palate functioning and voice loudness (all 5 out of 8 patients) in group I. **Table 2** shows the efficiency of the articulation organs functions in 12 ALS patients divided into groups measured 28 days after the application of LIN^-^ cell treatment compared to the baseline values (day 0). In group I improvement was found in 53.85% of the analyzed functions whereas the other 46.15% of them remained unimproved. In group II the analogous results were 35% and 65% respectively. It is noteworthy that tongue mobility was the only articulatory function that did not improve in all 12 patients.

**Table 3** demonstrates the CSF concentrations of trophic factors (NGF, BDNF, PDGF-AA, PEDF), angiogenic growth factors (VEGF-A, ANGP-2) and proinflammatory proteins (C3, C4, CRP, complement factor H) in group I and II of the ALS patients 28 days after the LIN^-^ cell administration. It is worth stressing that the initial C3 level in CSF was almost two times higher in group II (*p=*0.23). No statistically significant differences between the pre- and post-treatment concentrations of C3, C4 and CRP were observed in either of the two groups. On day 28 following the cell administration, we observed a declining tendency for the levels of CRP in the cerebrospinal fluid, particularly noticeable in group I (*p=*0.13). The baseline CSF concentration of BDNF and PDGF-AA was higher in group I, compared to group II (*p=*0.07 and *p=*0.11 respectively). Interestingly, in group I we observed a statistically significant decrease in concentrations of BDNF, ANGP-2 and PDGF-AA (*p=*0.03, *p=*0.03 and *p=*0.03 respectively), while VEGF-A displayed a linear downward trend 28 days after the cell transplantation procedure. No significant differences were observed in group II. On day 28, the CSF concentration of BDNF in group I fell to a similar level to the baseline in group II.

**Figure 1:**
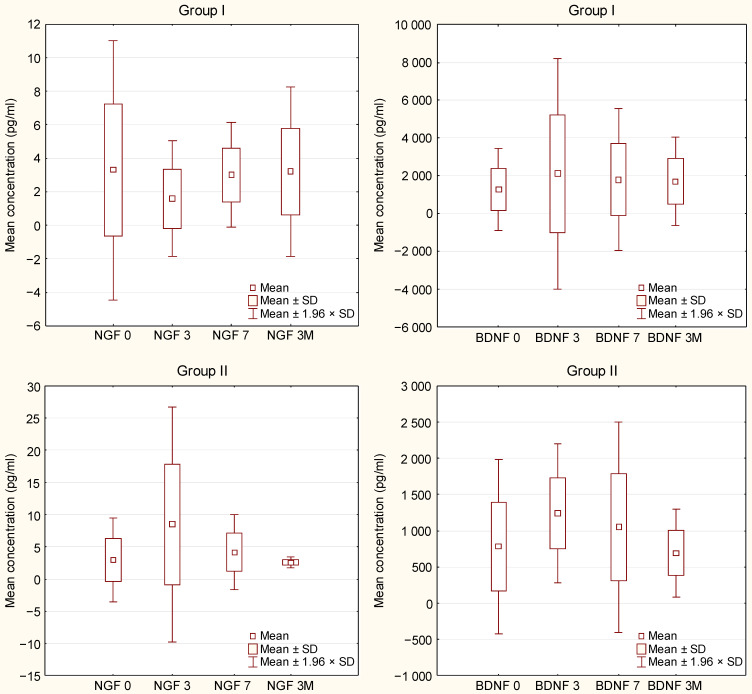
NGF and BDNF levels in blood plasma in groups I and II of the ALS patients on day 0, 3, 7, and 3 months after the LIN^-^ cell infusion (Friedman ANOVA & Kendall Concordance).

**Figure 1** displays plasma concentrations of BDNF and NGF at different time points following the LIN^-^ cell administration to group I and II of the ALS cohort. It was established that BDNF concentrations in both groups and NGF concentration in group II rise until they reach the highest levels 2-3 days following the injection, upon which they begin to decrease steadily on subsequent days. The differences were statistically unsignificant (group I: NGF *p=*0.99, BDNF *p=*0.46; group II: NGF *p=*0.2; BDNF *p=*0.95).

## Discussion

Dysarthric disorders, frequently afflicting ALS patients, adversely affect quality of life and lead to social isolation 25-26. Even the latest state-of-the-art technologies, utilized in highly specialized communicators including brain-computer interfaces, cannot replace interpersonal verbal communication. It has been demonstrated that alternative methods of communication are not entirely accepted by ALS patients due to the limited transfer of information they offer 8-9. Hence, the ongoing search for new therapeutic strategies that would enable early intervention at the root of the disease pathogenesis 27.

The application of stem/progenitor cells in ALS could improve the patients' motor functions or, at least, slow down the progression of neurodegenerative processes, which in turn would preserve the performance of the articulation organs. Broadly defined stem cells are thought to provide trophic support for the host neurons, decelerate neurogenerative process, produce deficient neurotransmitters and differentiate into progenitor oligodendroglial or neural precursor cells 10, 28.

In our study, we performed an autologous administration of bone marrow-derived lineage-negative stem/progenitor cells collected from ALS patients. The cells, isolated by a negative selection process, demonstrate far higher long-term self-regeneration potential and capacity to produce neurotrophic and angiopoietic factors than any other nucleated cells 20, 29. Our previous study showed that the LIN^-^ stem/progenitor cell administration is a safe procedure that could lead to possibly desirable outcomes such as an increased concentration of trophic factors as well as enhanced expression of different miRNA 11. Here, we aimed to investigate possible effect of LIN^-^ cells administration on articulatory functions, and simultaneously on trophic and pro-inflammatory factors.

Of the 12 ALS patients enrolled in this study, 8 (group I) demonstrated improved articulatory functions on the 28^th^ day since the cell administration, particularly noticeable in the phonation time, lips and soft palate performance, swallowing reflex and voice loudness. Interestingly, group I presented with slightly older age, later age of disease onset and shorter symptom duration than group II, although all these characteristics were barely outside the range of significance (*p=*0.07, 0.07 and 0.11, respectively). It may be partly explained with a shorter period of neurodegeneration process and a greater responsiveness to treatment. Additionally, so called immunological aging, which encompasses age-associated declines in the immune system (immunosenescence) 30 may play a significant role in severity of ALS course.

Improvement of swallowing reflex and soft palate performance among patients from group I might play a substantial role in mortality reduction. Impaired swallowing and reduced or absent cough reflex in ALS patients is a serious abnormality inevitably leading to the accumulation of excessive saliva in the airways, which adversely affect respiration. Muscle impairments resulting from damage to the medulla oblongata are responsible for weakened inspiration and expiration and impaired cough reflex. Cough effectiveness is suboptimal when peak cough flow (PCF) is less than 270 L/min 31. Accumulation of the mucus and other secretions that obstruct the airways can lead to partial or total collapse of the lung, and consequently to acute or acute-on-chronic respiratory failure, which directly accounts for death in most of ALS cases 32-34.

The important role of neuroinflammation mediated by glial and immune cells in the pathogenesis of ALS is widely accepted 35. Therefore, we have focused first on neuroinflammation in our ALS patient population, as numerous inflammatory proteins have been previously shown to be altered by this disease 36-37. The baseline CSF concentration of C3 was almost two times higher in group II of our study, comprising subjects who displayed no post-treatment articulation enhancement (*p=*0.23). Several studies in ALS mice models have correlated the increase in complement components with the disease progression 17-19, which might in part also explain our findings. Initial differences in C3 concentration may be result of younger age and longer disease duration in group II patients. Neither group I subjects, nor their group II counterparts, exhibited statistically significant differences in the concentrations of C3, C4 and CRP prior the stem cell transplantation and 28 days after its application. On day 28, we observed a declining trend in the concentration of CRP in the cerebrospinal fluid, which was particularly prominent in group I (statistically insignificant, *p=*0.13). The observation might suggest that LIN^-^ application could affect ongoing inflammation, presumably suppressing it 11. High baseline levels of proinflammatory factors appear to be an adverse prognostic indicator related to both the disease progression and the ability to control its course.

For many years, researchers dealing with the subject of ALS have focused on neurotrophic factors. Attempts to apply neurotrophins to slow down the progression of the disease have been repeatedly carried out both in humans and on animal models 35-37. As most neurotrophins present in body fluids have a very short half-life, any attempted treatments with recombined factors would be costly and the effects short-lived. Repeated cell injections performed at short intervals or administration of genetically modified stem cells would ensure prolonged and steady secretion of the desired soluble neurotrophic agents and create appropriate microenvironment for the neuroregenerative processes 14.

In our study, the baseline CSF concentration of BDNF was several times higher in group I, compared to group II (*p=*0.07). Similarly, PDGF-AA level at day 0 was about twice as high in group I than in group II (*p=*0.11). CSF concentrations of BDNF, PDGF-AA and ANGP-2 in group I displayed a significant decrease 28 days following LIN^-^ cell administration. On day 28, the CSF concentration of BDNF in group I fell to a similar level to the baseline in group II. It remains unclear why, in parallel with the improvement of articulation, a statistically significant decrease in neurotrophins concentration was observed. Perhaps these neurotrophins have been used for central nervous system (CNS) stimulation. It was previously described, that transplantation of adipose tissue-derived stem cells, modified muscle progenitor cells or neural progenitor cells in ALS mice provides neuroprotective effects by production of trophic factors (such as: BDNF, NGF, IGF-1 and VEGF) what delays disease progression, and prolongs the life span of ALS mice 38-40. It is likely that, due to the short half-life of trophic factors, CSF concentrations changed soon after cells administration. These presumptions seem to be reflected by the fact that the peak plasma concentrations of neurotrophins BDNF and NGF in our study were observed on the 3^rd^ day following LIN^-^ cell treatment. However, this issue still requires further in-depth research.

## Conclusions

Our experimental adjuvant therapy with the application of autologous bone marrow-derived lineage-negative cells injected intrathecally proved entirely safe for ALS patients. No immediate or delayed, topical or systemic adverse events following the treatment were observed. The short-termed post-treatment improvement of articulatory functions, particularly the phonation time, lips and soft palate efficacy, swallowing reflex and voice loudness, observed in most of the subjects provides valuable incentives for further comprehensive investigations. Parallel evaluation of trophic and proinflammatory factors casts new light at the extent of complexity characterizing etiopathogenesis of the ALS course.

## Study limitations

Our preliminary study provides a stimulating introduction to further investigations concerning the effect the application of the LIN^-^ stem cells has on the quality of speech in ALS patients. Undoubtedly, one of the study limitations was the considerable diversity regarding the age of the subjects (21-65) or duration of the disease (8 - 108 months). Also, the absence of a control group, which was dictated by ethical considerations, made it difficult to form reliable conclusions and establish whether the changes in relevant parameters were due to the injected cells themselves or a response of the host tissue. Additionally, a period of 28 days from transplantation may be too distant timepoint to assess actual changes in neurotrophins' concentrations 11. Our findings enabled us to pinpoint a number of parameters to be taken into consideration in larger cohort studies or while performing multiple LIN^-^ stem cells injections at various time points. Multiple injections of stem cells can be expected to potentially prolong beneficial effect of neurotrophins in CSF among ALS patients. Encouraged by the results of this pilot study and to address some above-mentioned drawbacks, we have set another experiment. 32 patients with sporadic ALS underwent autologous LIN^-^ stem/progenitor cell intrathecal administration. Patients were examined for articulatory functions by means of VHI questionnaire and FDA on days 0, 7 and 28 following the LIN^-^ cell administration. In parallel, we carried out the analysis of selected trophic (BDNF, NT-3, NGF-β) and proinflammatory factors (TNF-α, TNF-R, CRP) in CSF in day 0 and on the 7^th^ day post injection. Obtained results have been already published 41. We are currently conducting research on even larger cohort of ALS subjects with 3 consecutive LIN^-^ cell administrations with a one-year follow-up period.

## Figures and Tables

**Table 1:** Characteristics of the groups

Characteristic	Group I (n=8)	Group II (n=4)	p value
Age (mean ± SD, years)	55.88 ± 9.28	39.25 ± 14.34	0.07^a^
Sex (male/female)	5/3	3/1	0.83^b^
Symptom duration (mean ± SD, months)	31.25 ± 31.98	55.00 ± 23.47	0.11^a^
Age at disease onset (mean ± SD, years)	53.13 ±10.70	34.75 ±14.43	0.07^a^
Disease onset (bulbar/limb)	3/5	1/3	0.27^b^
ALS-FRSr score before LIN^-^ Tx (mean ± SD)	19.00 ± 6.60	23.75 ± 7.14	0.27^a^
Norris scale score before LIN^-^ Tx (mean ± SD)	66.25 ± 14.22	75.75 ± 20.06	0.44^a^
Number of LIN^-^ cells administered (mean ± SD)	(8.99 ± 6.65)×10^6^	(8.98 ± 4.37)×10^6^	1.00^a^

a: U Mann-Whitney analysis of variance by ranks for more than two groups. b: chi-squared test or Yates's chi-squared test.

**Table 2:** Efficiency of the articulation organs in groups I and II of the ALS patients 28 days after the administration of LIN^-^ cells

Articulatory functions	Group I (n=8)			Group II (n=4)			
	deterioration	stabilization	improvement		deterioration	stabilization	improvement
Breathing	0	4	4		0	4	0
Cough reflex	0	4	4		0	2	2
Tongue mobility	2	6	0		0	4	0
Swallowing reflex	0	3	5		1	3	0
Lips performance	0	2	6		0	2	2
Palate performance	0	3	5		0	2	2
Voice loudness	0	3	5		0	1	3
Pitch	0	4	4		0	1	3
Phonation time	0	1	7		0	3	1
Control of saliva	0	4	4		0	3	1
**Total**	**2**	**34**	**44**		**1**	**25**	**14**
							

**Table 3:** CSF concentrations of trophic factors, angiogenic growth factors and proinflammatory proteins in group I and II of the ALS patients 28 days after the LIN^-^ cell infusion; * *p<*0.05; 0d vs 28d (Wilcoxon matched-pairs test for two groups); * *p<*0.05 Group I vs Group II (Mann-Whitney *U* test).

CSF concentrations of trophic factors and proinflammatory proteins on day 0 and 28.	Group I (n=8)		Group II (n=4)		Group I vs Group II
	Mean concentration ± SD (pg/ml)	*p* value	Mean concentration ± SD (pg/ml)	*p* value	*p* value
CRP_0d	8.45 ± 5.74	0.13	9.22 ± 8.50	0.47	0.79
CRP_ 28d	4.72 ± 4.75		5.69 ± 9.55		0.69
C4 _0d	122.70 ± 52.44	0.18	94.72 ± 29.19	0.72	0.53
C4_28d	141.04 ± 65.04		102.73 ± 8.06		0.65
C3_0d	384.38 ± 169.55	0.24	636.50 ± 263.43	0.27	0.23
C3_28d	447.38 ± 184.88		457.00 ± 334.74		0.79
NGF_0d	0.32 ± 0.17	0.50	0.23 ± 0.19	0.59	0.79
NGF_28d	0.32 ± 0.19		0.29 ± 0.06		0.93
BDNF_0d	34.46 ± 80.00	*0.03	1.88 ± 2.51	0.14	0.07
BDNF_28d	1.61 ± 4.33		0.21 ± 0.43		0.93
ANGP-2_0d	107.86 ± 38.19	*0.03	110.74 ± 41.89	0.72	0.93
ANGP-2_28d	84.69 ± 27.96		117.50 ± 64.20		0.65
VEGF-A_0d	10.45 ± 6.63	0.09	10.15 ± 9.79	0.98	0.93
VEGF-A_28d	6.07 ± 3.36		9.05 ± 12.84		1.00
PDGF-AA _0d	9.44 ± 7.26	*0.03	4.58 ± 0.31	0.07	0.11
PDGF-AA_28d	4.22 ± 0.77		5.38 ± 0.76		0.07
PEDF_0d	15.29 ± 4.41	0.61	21.02 ± 5.30	0.07	0.07
PEDF_28d	14.71 ± 4.25		14.74 ± 4.54		0.93
COMP-FH_0d	1477.00 ± 520.80	0.61	1084.75 ± 294.83	0.72	0.53
COMP-FH_28d	1242.63 ± 510.19		1142.25 ± 401.03		0.79
